# Treatment Outcomes in Septic Arthritis of the Foot and Ankle in People Who Inject Drugs

**DOI:** 10.1177/2473011420928893

**Published:** 2020-07-31

**Authors:** Margaret V. Shields, Alexander Toppo, Mariano E. Menendez, David Tybor, Peter Dewire, Alysse Wurcel, Matthew Salzler

**Affiliations:** 1 Department of Orthopedics, Tufts Medical Center, Boston, MA, USA; 2 Tufts University School of Medicine, Boston, MA, USA; 3 Division of Geographic Medicine and Infectious Diseases, Department of Medicine, Tufts Medical Center, Boston, MA, USA; 4 Division of Sports Medicine, Tufts University School of Medicine, Tufts Medical Center, Boston, MA, USA

**Keywords:** septic arthritis, sepsis, intravenous drug use, substance abuse disorder, treatment outcomes

## Abstract

**Background:**

Although injection drug use (IDU) is a known risk factor for septic arthritis (SA) of the foot and ankle (F&A), disease and hospitalization outcomes are poorly characterized. We evaluated national trends, demographic characteristics, and hospitalization outcomes of SA of the F&A in people who inject drugs vs those who do not.

**Methods:**

Using the Nationwide Inpatient Sample, we identified all patients aged 15-64 with a principal discharge diagnosis of SA of the F&A from 2000 to 2013 and evaluated if they were related or unrelated to IDU. We assessed differences in demographic characteristics and in-hospital outcomes in these groups.

**Results:**

From 2000 to 2013, there were an estimated 14,198 hospitalizations for SA of the F&A in the United States, and 11% were associated with IDU (SA-IDU). Compared to SA unrelated to IDU, people with SA-IDU were significantly more likely to be younger, black, and have Medicaid or no insurance. People with SA-IDU were significantly more likely to leave against medical advice (9.7% vs 1.4%, *P* < .001), have a longer length of stay (9.2 vs 6.8 days, *P* < .001), and incur increased hospital charges ($58 628 vs $38 876, *P* = .005). People with SA-IDU were significantly less likely to receive an arthroscopy (1.5% vs 6.5%, *P* < .001) or arthrotomy (2.2% vs 11.0%, *P* < .001) of the foot.

**Conclusion:**

People with SA-IDU of the F&A had suboptimal hospitalization outcomes with greater costs. Recognizing risk factors and proactively addressing potential complications of substance use disorder in the hospital should be prioritized by the orthopedic community.

**Level of Evidence:**

Level III, retrospective cohort study.

## Introduction

Septic arthritis (SA) requires urgent treatment to prevent irreversible cartilage destruction, which can occur within hours.^12^ Prompt diagnosis of SA of the foot and ankle is challenging as it can present similar to other infectious and inflammatory conditions of the foot and ankle such as cellulitis, osteomyelitis, Charcot neuroarthropathy, and gout.^2^ If using unsterile techniques, people who inject drugs (PWID) are at risk for developing SA when using the veins of their foot and ankle. These veins in the foot and ankle may be chosen for injection because of several reasons, including relatively easy access and ability to conceal the injection site.^11^ In light of the opioid epidemic, bacterial infections like endocarditis, septic arthritis, and epidural abscesses are increasing.^3,10^

Little is known about the epidemiologic trends, demographics, and outcomes of SA of the foot and ankle in PWID compared with people who do not inject drugs. In this study, we addressed the following questions: (1) How many patients are admitted annually with septic arthritis of the foot and ankle and what percentage of these patients use injection drugs? (2) Are there demographic trends for patients with injection drug use related to septic arthritis of the foot and ankle from 2000 to 2013? (3) How do rates of complications, operations, length of stay, and leaving against medical advice (AMA) compare between patients with foot and ankle SA-IDU and those without injection drug use history? (4) Does the presence of concurrent cellulitis affect treatment and outcomes in PWID vs people who do not inject drugs?

## Methods

Data for this study were obtained from the Healthcare Cost and Utilization Project Nationwide Inpatient Sample (NIS). Briefly, the NIS is the largest publicly available patient database, comprising a 20% random stratified sample of all inpatient discharges in the United States.^1^ The database includes demographic information, procedural and diagnosis codes (Classification of Diseases, Ninth Revision, Clinical Modification [ICD-9-CM]), and clinical outcomes data such as length of stay, total charges, and disposition. National estimates for all US hospitalizations can be made by applying the provided sample weights to the data set.

We identified cases discharged with a principal diagnosis of septic arthritis of the ankle and foot (711.07) between 2000 and 2013. We limited our analysis to cases in patients aged 15-64 years and excluded patients with a history of ankle arthroplasty (V43.66). We used previously published algorithms to classify septic arthritis cases with IDU (IDU-SA) as those with a diagnosis code for illicit drug use or hepatitis C virus (70.40, 70.44, 70.51, 70.54, 70.70, 70.71, V02.62), and septic arthritis cases without IDU (non-IDU-SA) as those without these diagnosis codes (Appendix 1).^10,14^ Sample weights were applied as described in the NIS user guide to obtain national estimates and 95% confidence intervals for all calculations described herein.^4,5^ As per AHRQ guidelines, hospital charges were adjusted to the 2013 U.S. Gross Domestic Product.^4,5^

The adjusted Wald test was used to measure differences in demographics between SA cases with and without IDU, and multivariable linear regressions adjusted for age, race, and gender were employed to analyze trends in these characteristics over time. We measured clinical and hospital outcomes such as length of stay, leaving against medical advice, mortality, and hospital charges in the 2 groups and applied the adjusted Wald test to determine differences between them. The same procedure was used to compare frequency of diabetes mellitus (250.00-250.93) and peripheral vascular disease (443.9) among IDU-SA and non-IDU-SA cases.

We applied multivariable logistic regressions adjusted for age, race, gender, diabetes mellitus, and peripheral vascular disease to determine the odds ratios (ORs) of receiving the following procedures for patients with IDU-SA vs those within non-IDU-SA: arthroscopy of the ankle (80.27, 80.47, 80.77, 80.87, 80.97) and arthroscopy of the foot and toe (80.28, 80.48, 80.78, 80.88, 80.98), arthrotomy of the ankle (80.17) and arthrotomy of the foot and toe (80.18), and arthrocentesis (81.91). We evaluated whether there were differences in the proportion of patients who received an ankle fusion (81.11), had a toe amputation (V49.72), foot amputation (V49.73), ankle amputation (V49.74), or below-knee amputation (V49.75) between the 2 groups.

We then evaluated IDU-SA and non-IDU-SA cases for any differences between frequency of cellulitis of the ankle (682.6), cellulitis of the foot (682.7), and cellulitis of the toe (681.10). To determine whether cellulitis was an effect modifier for clinical outcomes among those with septic arthritis, we ran similar adjusted linear and logistic regressions for IDU-SA and non-IDU-SA patients with and without cellulitis of the foot, toe, or ankle.

We conducted all analyses with Stata, version 15 (StataCorp, College Station, TX), and set significance at a 2-sided alpha of 0.05. Figures were created using Microsoft Excel 16. As our study used deidentified data, it was granted an exemption by the Tufts Health Sciences Institutional Review Board.

## Results

### Number of Patients With Septic Arthritis Related to IDU

**Figure 1.**
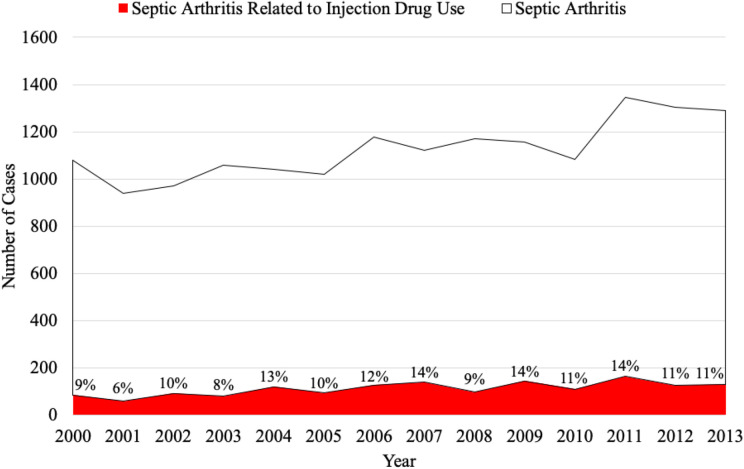
The total number of septic arthritis of the foot and ankle cases stratified by injection drug use (Nationwide Inpatient Sample, 2000-2013) is shown. Percentages represent the proportion of annual septic arthritis cases related to injection drug use.

We identified 14 198 patients with SA of the foot and ankle from 2000 to 2013 (Table 1, Appendix 2, Figure 1). Of this group, 12 625 (89%) were patients with non-IDU-SA, whereas 1558 (11%) were patients with IDU-SA. The overall incidence of SA of the foot and ankle in the general population over this time frame remained relatively stable, starting at 0.53/100 000 person-years (95% CI, 0.44, 0.63) in 2000 and increasing to 0.55/100 000 person-years (95% CI, 0.48, 0.63) in 2013. Similarly, the incidence of septic arthritis related to IDU also remained stable over the 13 years, starting at 0.05/100 000 person-years (95% CI, 0.02, 0.07) in 2000 and ending at 0.06/100 000 person-years (95% CI, 0.04, 0.09) in 2013. Although the incidence did not significantly change, the percentage of total patients with SA of the F&A who have a history of IDU has steadily increased from 8.5% (95% CI, 5.3, 13.6) in 2000 to 11.2% (95% CI, 7.8, 15.9) in 2013.

**Table 1.** Demographic Measurements for Patients With Septic Arthritis of the Foot and Ankle in the United States, Stratified by Injection Drug Use: 2000-2013 Combined, Nationwide Inpatient Sample.^a^

Indicator	All septic arthritis, n (%)	Septic arthritis with Injection drug use, n (%)	Septic Arthritis Without Injection Drug Use, n (%)	*P* Value
Patients, Total Weighted Number	14 198 (100)	1558 (11)	12 625 (89)	
Age, y				
15-34	2954 (21)	320 (21)	2634 (21)	.90
35-54	7089 (50)	**910 (58)**	**6178 (49)**	**.001**
55-64	4140 (29)	**328 (21)**	**3812 (30)**	**<.001**
Sex				
Male	9718 (69)	8668 (67)	1050 (69)	
Female	4458 (31)	508 (33)	3949 (31)	.65
Race				
White	7705 (54)	771 (50)	6933 (55)	.10
Black	1725 (12)	**298 (19)**	**1427 (11)**	**.0016**
Hispanic	1322 (9)	160 (10)	1163 (9)	.60
Asian or Pacific Islander	174 (1)	15 (1)	159 (1)	.63
Native American	137 (1)	15 (1)	122 (1)	.99
Other	331 (2)	29 (2)	302 (2)	.50
Primary insurance				
Medicare	2179 (15)	246 (16)	1933 (15)	.81
Medicaid	2164 (15)	**581 (37)**	**1579 (13)**	**<.001**
Private insurance	6887 (49)	**286 (18)**	**6591 (52)**	**<.001**
Self-pay	1563 (11)	**293 (19)**	**1270 (10)**	**<.001**
No charge	274 (2)	63 (4)	211 (2)	.101
Other	1082 (8)	89 (6)	993 (8)	.14

^a^ Adjusted Wald test. Bolded text indicates statistically significant results.

### Age, Racial Trends, and Insurance Types

Demographic analysis revealed that IDU-SA patients were significantly more likely to be 35-54 years old (58% vs 49%, *P* = .001), to be black (19% vs 11%, *P* = .002), and to have Medicaid (37% vs 13%, *P* < .001) or self-pay (19% vs 10%, *P* < .001) than non-IDU-SA patients (Table 1).

### Complications, Reoperations, Leaving Against Medical Advice, and Length of Stay

Patients with IDU-SA left AMA 9.7% of the time vs non-IDU-SA patients who left AMA 1.4% of the time (adjusted odds ratio 8.00, 95% CI 4.59-13.96, *P* < .001) (Tables 2 and 3). Patients with IDU-SA had significantly longer LOS than patients with non-IDU-SA (9.2 vs 6.8 days, *P* < .001). In addition, patients with IDU-SA were significantly more likely to have an associated foot and ankle cellulitis than non-IDU-SA patients (44.3% vs 35.1%, adjusted odds ratio 1.56, 95% CI, 1.19-2.03, *P* = .001).

**Table 2.** Association of Injection Drug Use With Hospitalization Outcomes Among Patients With and Without Septic Arthritis of the Foot and Ankle, United States: 2000-2013, Nationwide Inpatient Sample.^a^

Hospital outcome	AOR (95% CI)	*P* Value^b^
Left against medical advice	**8.00 (4.59, 13.96)**	**<.001**
Foot		
Received arthroscopy	**0.31 (0.12, 0.77)**	**.012**
Received repeat arthroscopy	N/A	N/A
Received arthrotomy	**0.23 (0.11, 0.51)**	**<.001**
Received repeat arthrotomy	N/A	N/A
Ankle		
Received arthroscopy	0.89 (0.64, 1.23)	.49
Received repeat arthroscopy	0.74 (0.43, 1.26)	.27
Received arthrotomy	1.24 (0.91, 1.69)	.18
Received repeat arthrotomy	1.00 (0.42, 2.36)	.99
Received arthrocentesis	1.17 (0.87, 1.57)	.30
Received repeat arthrocentesis	1.01 (0.47, 2.18)	.99
Received ankle fusion	0.58 (0.18, 1.89)	.36
Had cellulitis of the ankle, foot, or toe	**1.56 (1.19, 2.03)**	**.001**
Had diabetes mellitus	0.92 (0.68, 1.23)	.57
Had peripheral vascular disease	1.99 (0.98, 4.02)	.06
Died	2.55 (0.47, 13.84)	.28

Abbreviations: AOR, adjusted odds ratio; CI, confidence interval.

^a^ Bolded text indicates statistically significant results.

^b^ Multivariable logistic regression adjusted for age, sex, race, diabetes mellitus, and peripheral vascular disease.

**Table 3.** Hospitalization Outcomes for Patients With Septic Arthritis of the Foot and Ankle in the United States, Stratified by Injection Drug Use: 2000-2013 Combined, Nationwide Inpatient Sample.

Hospitalization Outcomes	Patients With Injection Drug Use^a^	Patients Without Injection Drug Use^a^	Adjusted Difference (Injection Drug Use–No Drug Use)^a^	*P* Value^b^
Length of stay (d)	9.2 (8.3, 10.1)	6.8 (6.5, 7.1)	**1.88 (0.86, 2.91)**	**<.001**
Total charge—unadjusted for inflation (US $)	49 937 (42 034, 57 841)	34 878 (32 970, 36 786)	**12 759 (4448, 21 070)**	**.003**
Total charge—adjusted for inflation (2013 US $)	54 531 (46 056, 63 005)	38 482 (36 401, 40 563)	**13 261 (4565, 21 956)**	**.003**
Left against medical advice (%)	9.7 (6.9, 13.5)	1.4 (1.0, 2.0)	**9.0 (5.3, 12.8)**	**<.001**
Diagnosed with cellulitis of the ankle, foot, or toe (%)	44.3 (38.9, 50.0)	35.1 (33.2, 37.0)	**10.4 (4.0, 16.9)**	**.002**
Diagnosed with diabetes mellitus (%)	27.8 (23.2, 32.9)	29.2 (27.5, 31.0)	–2.1 (–7.5, 3.4)	.45
Diagnosed with peripheral vascular disease (%)	3.4 (1.9, 6.0)	2.1 (1.6, 2.7)	1.7 (–0.6, 4.0)	.14
Foot				
Received arthroscopy (%)	1.5 (0.6, 3.6)	6.5 (5.6, 7.6)	**–3.9 (–5.8, –2.0)**	**<.001**
Received repeat arthroscopy^‡^ (%)	0	0.9 (0.6, 1.4)	–0.7 (–1.1, –0.4)	<.001
Received arthrotomy (%)	2.2 (1.0, 4.5)	11.0 (9.8, 12.3)	**–7.7 (–10.1, –5.3)**	**<.001**
Received repeat arthrotomy (%)	0	0.4 (0.2, 0.7)	–0.3 (–0.6, –0.1)	.010
Ankle				
Received arthroscopy (%)	21.6 (17.3, 26.6)	21.9 (20.3, 23.5)	–1.9 (–6.9, 3.2)	.47
Received repeat arthroscopy (%)	5.4 (3.4, 8.4)	6.8 (5.9, 7.8)	–1.7 (–4.3, 0.9)	.21
Received arthrotomy (%)	28.7 (23.6, 34.4)	0.23 (0.21, 0.25)	3.9 (–2.0, 9.8)	.20
Received repeat arthrotomy (%)	2.5 (1.3, 4.8)	2.2 (1.7, 2.9)	<–0.1 (–1.9, 1.9)	.98
Received arthrocentesis (%)	27.9 (23.1, 33.3)	23.5 (21.8, 25.2)	2.9 (–2.8, 8.7)	.32
Received repeat arthrocentesis (%)	2.5 (1.2, 4.9)	2.3 (1.8, 2.9)	<0.1 (–1.9, 2.0)	.98
Received ankle fusion (%)	1.2 (0.5, 3.3)	1.8 (1.2, 2.5)	–0.8 (–2.2, 0.6)	.28
Had below-the-knee amputation (%)	1.2 (0.5, 3.2)	1.7 (1.3, 2.3)	–0.9 (–2.3 to 0.5)	.21
Mortality (%)	0.9 (0.3, 2.8)	0.3 (0.1, 0.6)	0.4 (–0.6 to 1.4)	.44

^a^ Numbers in parentheses indicate 95% confidence intervals.

^b^ Adjusted for age, gender, race, diabetes mellitus, and peripheral vascular disease. Bolded text indicates statistically significant results.

People with IDU-SA were significantly less likely to receive operative intervention of the foot compared to people with non-IDU-SA including arthroscopy (1.5 vs 6.5%, *P* < .001) and arthrotomy (2.2 vs 11.0%, *P* < .001). There was no significant difference in patients receiving arthroscopy or arthrotomy of the ankle or arthrocentesis.

Patients who inject drugs were significantly more likely to have a concurrent diagnosis of cellulitis of the F&A, with a rate of 44.3% (38.9-50.0) in IDU-SA patients vs 35.1% (33.2-37.0) in non-IDU-SA patients (*P* = .002). People with IDU-SA with associated cellulitis of the ankle, foot, and toe were significantly less likely than non-IDU-SA with associated cellulitis to undergo a transtibial amputation (0.7% vs 2.3%, *P* < .001). Notably, there was no significant difference in the number of people with IDU-SA vs non-IDU-SA diagnosed with DM (27.8% vs 29.2%, *P* = .45) or PVD (3.4% vs 2.1%, *P* = .14).

### Hospital Charges

**Figure 2.**
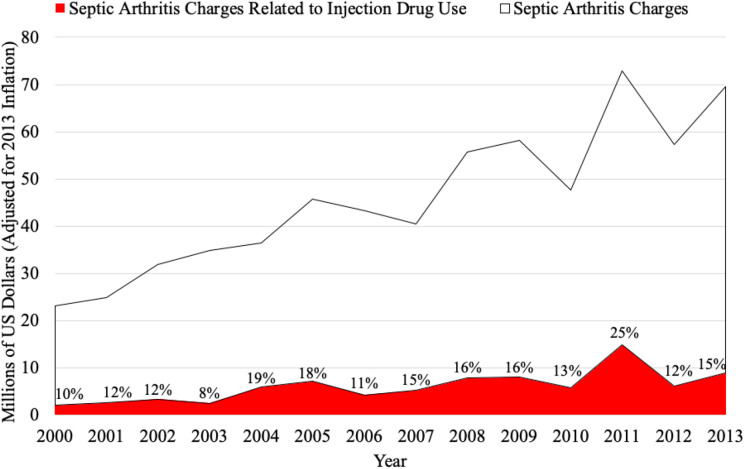
The total hospital charges of septic arthritis of the foot and ankle stratified by injection drug use (Nationwide Inpatient Sample, 2000-2013) are shown. Percentages represent the proportion of annual septic arthritis charges related to injection drug use.

The total hospital charge for people presenting with IDU-SA, adjusted for inflation, has significantly increased from 2000 to 2013 (Appendix 2, Figure 2). In year 2000, the average cost was $23 868 (95% CI, 15 438, 32 298), whereas in 2013 the average cost increased to $68 327 (95% CI, 41 121, 95 532, *P* = .001). People with IDU-SA had significantly higher total hospital charges than patients with non-IDU-SA ($54 351 vs $38 482, *P* = .003).

## Discussion

Over the 14 years of our study, the percentage of patients diagnosed with SA of the foot and ankle that was specifically related to intravenous drug use steadily increased from 8.5% of all F&A SA cases in 2000 to 11.2% in 2013. Our results add to the available literature on the increasing impact the nationwide opioid epidemic is having across the health care system.

Septic arthritis is a medical emergency that is known to be associated with significant morbidity and mortality.^8,10^ In addition to irreversibly damaging the joint itself, untreated septic arthritis can spread the infection systemically, leading to severe problems such as epidural abscesses, infective endocarditis, and sepsis.^8,10^ Mathews et al reported an 11% mortality rate in patients presenting with monoarticular septic arthritis, and the percentage increases as more joints are involved.^8^ Although septic arthritis of the foot and ankle represents only about 3% to 7% of all septic arthritis cases, early identification and treatment remain equally as important as septic arthritis in other joints.^2^ Infections of the foot and ankle are associated with substantial morbidity, disability, and even the potential for limb loss.^2^ In the younger patient population that is typically affected from IDU-SA of the foot and ankle, it is essential to expeditiously diagnose and initiate early treatment given potential lifelong consequences associated with delayed diagnosis and treatment. Based on our results, clinicians should have a heightened awareness of the demographic that is more commonly affected from IDU-SA of the foot and ankle. Specifically, this includes patients aged 35-54 years, patients who are black, and patients who have Medicaid or self-pay for insurance. A high clinical suspicion in all patients, but specifically in this demographic, may aid in early diagnosis and treatment to decrease long-term complications.

The foot and the ankle are common injection sites for IV drugs because of access provided by the dorsal venous arch and greater saphenous vein.^11^ A recent study noted that PWID who are found to have septic arthritis of the leg have higher rates of death during hospitalization, higher chances of repeat operations, more resource utilization, and higher probability of leaving AMA.^10^ Considerations for patients with SA of the foot and ankle specifically include identification of systemic conditions such as diabetes, peripheral vascular disease, chronic liver or kidney disease, immunosuppressive status, and gout. The mainstay of treatment of septic arthritis of any joint, including within the foot and/or ankle, is arthrotomy, either arthroscopically or open, with irrigation and debridement of the joint space.

We found that patients presenting with SA of the foot and ankle associated with IDU were significantly more likely to leave the hospital AMA but also on average had a longer LOS. It has been postulated in previous studies that PWID have a higher likelihood than the general population of leaving AMA because of inadequate pain management during hospitalization, withdrawal symptoms, and/or desire to maintain their drug use habits.^7,13^ These studies highlight the importance of multimodal management with an addiction specialist involved in the care of the patient to decrease the likelihood of the patient leaving AMA. In addition, research has shown that PWID are more likely to receive appropriate medication and complete antibiotic therapy if an addiction psychiatrist is consulted, emphasizing the importance of prioritizing this early during hospitalization.^7^ However, despite the evidence that 10% of PWID with SA of the foot and ankle leave AMA, the average hospital stay is still significantly longer than their non-IDU counterparts. Several factors may have contributed to the increased LOS among IDU-SA patients. Septic arthritis patients who inject drugs may potentially be in poorer health than their non-IDU counterparts and require more treatment. Additionally, if a central line for IV antibiotics is placed, clinicians may elect to discharge IDU-SA patients after treatment is complete to avoid IDU through this route. Finally, social determinants of health such as lack of housing or the need for more comprehensive discharge planning may also contribute to longer hospital stays.

Despite the significantly longer length of stay for patients with IDU-SA of the foot and ankle, we found that these patients were significantly less likely to receive operative intervention on the foot than patients with non-IDU-SA, but no more or less likely to receive operative intervention of the ankle. Similarly, we found that IDU-SA patients with associated cellulitis of the ankle, foot, and toe were significantly less likely than non-IDU-SA with associated cellulitis to have a transtibial amputation. The percentage of patients with a diagnosis of diabetes mellitus or peripheral vascular disease was not significantly different between the 2 groups. The reduced numbers of operative interventions on patients with IDU-SA may be due to a variety of factors. Our research found that patients with IDU-SA had a significantly higher likelihood of having a concurrent cellulitis of the ankle, foot, or toe, so it is possible that the overlying cellulitis delayed diagnosis of the underlying SA in PWID. In addition, less surgical intervention may be attributed to refusal of treatment from the patients with IDU or unequal treatment of SA in patients with and without IDU. Thus, we propose that providers should have high suspicion of SA in those at risk for IDU-SA of the foot and ankle, especially when there may be a concurrent cellulitis, so that identification, management, and operative intervention for the SA are not delayed in this subgroup.

One of the major strengths of this study is the immense sample size that was obtained through the nationally representative NIS database. The large sample size in the database and applicability to the nation make this a valuable study that is relevant for the entire United States. In addition, the large sample size allows for comparisons that would not be possible in a nondatabase study. Additionally, literature for both generalized septic arthritis of the foot and ankle and IDU-related SA of the foot and ankle is very limited. This is the largest study that we know of that evaluates septic arthritis of the foot and ankle.

This study has a few limitations. The classification of injection drug use is based on a previously published algorithm of using the NIS database diagnosis code for illicit drug use or hepatitis C virus. Although there is a strong association of hepatitis C virus in approximately 80% of persons with IDU, this algorithm may underestimate the impact and magnitude of the problem because of the challenge in identifying all patients with IDU.^6^ Physicians diagnose IDU with modest accuracy and are often mislead by subjective cues. Even in familiar patients with a known history of IDU, practitioners have difficulty identifying active IDU. Although we may have misclassified a minority of patients with hepatitis C and no IDU as people who inject drugs, many more patients who injected drugs were likely misclassified as not injecting drugs.^9^ In addition, the NIS data set is based on data input from health care systems and depends on accurate data collection and recording. The race of patients in the NIS data set is identified by clinicians and not by patients themselves, possibly leading to additional errors. Lastly, the NIS data set does not follow patients through time so no follow-up data can be obtained. In the future, it would be beneficial to have studies that follow prospective cohorts of patients with IDU and non-IDU to evaluate the risk of developing SA and determine long-term outcomes.

In conclusion, research has shown that the number of individuals using opioids worldwide continues to rise and simultaneously the number of people primarily using the IV route for administration is increasing.^6^ The outcomes of this study reveal that IDU-SA of the foot and ankle is associated with suboptimal hospitalization outcomes and greater hospital resource utilization than non-IDU-SA of the foot and ankle. Using the results of this study and review of previous research, we suggest that going forward the following measures should be taken into consideration in order to improve hospitalization outcomes for PWID with SA of the foot and ankle. Providers should maintain a high suspicion of IDU-SA of the foot and ankle based on demographic analysis and clinical presentation. Providers should have a heightened awareness of the possibility of concurrent cellulitis and SA in PWID so that diagnosis and subsequent management of SA is not delayed. Providers should proactively address potential issues relating to substance use disorder early during hospital admission, and a team-based approach should be implemented with early consultation to addition psychiatry and infectious disease specialists.

## Supplemental Material

Supplemental Material, FAO928893-ICMJE - Treatment Outcomes in Septic Arthritis of the Foot and Ankle in People Who Inject Drugs Click here for additional data file. Supplemental Material, FAO928893-ICMJE for Treatment Outcomes in Septic Arthritis of the Foot and Ankle in People Who Inject Drugs by Margaret V. Shields, Alexander Toppo, Mariano E. Menendez, David Tybor, Peter Dewire, Alysse Wurcel and Matthew Salzler in Foot & Ankle Orthopaedics

## Appendix

**Appendix 1.** International Classification of Diseases, 9th Revision, Clinical Modification (ICD-9 CM) Diagnosis Codes for Identification of Injection Drug Use^a^

Condition	ICD-9 CM diagnosis code	ICD-9 CM procedure code	Diagnosis description
Illicit drug use	292.0		Drug withdrawal
	304.00-304.03		Opioid type dependence—unspecified, continuous, episodic, in remission
	304.20-304.23		Cocaine dependence—unspecified, continuous, episodic, in remission
	304.40-304.43		Amphetamine and other psychostimulant dependence—unspecified, continuous, episodic, in remission
	304.70-304.73		Combinations of opioid-type drug with any other drug dependence—unspecified, continuous, episodic, in remission
	305.5		Nondependent opioid abuse
	305.50-305.53		Opioid abuse—unspecified, continuous, episodic, in remission
	305.60-305.63		Cocaine abuse—unspecified, continuous, episodic, in remission
	305.70-305.73		Amphetamine or related acting sympathomimetic abuse—unspecified, continuous, episodic, in remission
	648.33		Drug dependence of mother, antepartum condition, or complication
	965.00		Poisoning by opium (alkaloids), unspecified
	965.01		Poisoning by heroin
	965.02		Poisoning by methadone
	969.7		Poisoning by psychostimulants
	E850.0		Accidental poisoning by heroin
	E850.1		Accidental poisoning by methadone
	E854.2		Accidental poisoning by other opiates and related narcotics
		94.45	Drug addiction counseling
		94.54	Referral for drug addiction rehabilitation
		94.64	Drug rehabilitation
		94.65	Drug detoxification
		94.66	Drug rehabilitation and detoxification
		94.67	Combined alcohol and drug rehabilitation
		94.68	Combined alcohol and drug detoxification
		94.69	Combined alcohol and drug rehabilitation and detoxification
Hepatitis C virus	070.41		Acute hepatitis C with hepatic coma
	070.44		Chronic hepatitis C with hepatic coma
	070.51		Acute hepatitis C without mention of hepatic coma
	070.54		Chronic hepatitis C without mention of hepatic coma
	707.00		Pressure ulcer, unspecified site
	707.01		Pressure ulcer, elbow
	707.03		Pressure ulcer, lower back
	707.10		Ulcer of lower limb, unspecified
	707.11		Ulcer of thigh
	707.12		Ulcer of calf
	707.13		Ulcer of ankle
	V02.62		Hepatitis C carrier

^a^ Cases with a diagnosis or procedural code for either illicit drug use or hepatitis C virus were classified as septic arthritis with injection drug use.

## Appendix

**Appendix 2.** Annual Trends in Demographics Among Patients With and Without Septic Arthritis of the Foot and Ankle With Injection Drug Use in the United States: Nationwide Inpatient Sample, 2000-2013.

Demographic Indicator	2000	2003	2006	2009	2012	2013	*P* Value for Trend	Coefficient (per Year)
SA national estimate	993 (828, 1158)	978 (798, 1158)	1050 (881, 1220)	1010 (817, 1203)	1180 (1020, 1340)	1160 (1003, 1317)		
IDU-SA national estimate	85 (41, 129)	79 (42, 117)	127 (70, 184)	144 (82, 207)	125 (74, 176)	130 (80, 180)		
SA incidence per 100 000 population	0.53 (0.44, 0.62)	0.51 (0.41, 0.60)	0.52 (0.44, 0.61)	0.50 (0.40, 0.59)	0.56 (0.49, 0.64)	0.55 (0.48, 0.63)		
IDU-SA incidence per 100 000 population	0.05 (0.02, 0.07)	0.04 (0.02, 0.06)	0.06 (0.03, 0.09)	0.07 (0.04, 0.10)	0.06 (0.04, 0.08)	0.06 (0.04, 0.09)		
IDU-SA (%)	8.6 (5.3, 13.6)	8.1 (5.1, 12.6)	12.1 (8.0, 17.9)	14.3 (9.9, 20.3)	10.6 (7.2, 15.4)	11.2 (7.8, 15.9)		
SA mean charge—adjusted for inflation (2013 US $)	22 446 (18 321, 26 571)	33 786 (27 782, 39 789)	37 859 (30 707, 45 011)	50 272 (38 301, 62 243)	44 585 (39 898, 49 273)	53 381 (47 300, 59 462)	**<.001**	1789
IDU-SA mean charge—adjusted for inflation (2013 US $)	23 868 (15 438, 32 298)	33 508 (18 779, 48 236)	34 793 (24 309, 45 276)	55 326 (43 250, 67 402)	48 534 (34 181, 62 887)	68 327 (41 121, 95 532)	**.001**	3134
Age group distribution (%) among patients with IDU-SA (years)								
Mean	44 (40, 48)	47 (43, 51)	41 (36, 46)	46 (42, 51)	47 (43, 51)	42 (37, 46)	.96	-0.9
15-34	12 (3, 35)	11 (3, 34)	35 (19, 56)	23 (10, 42)	20 (9, 40)	42 (25, 62)	.17	0.8
35-54	88 (65, 97)	67 (43, 85)	46 (29, 64)	53 (35, 71)	56 (36, 74)	35 (19, 54)	**.015**	–1.8
55-64	–	22 (8, 47)	19 (8, 38)	24 (12, 44)	24 (11, 44)	23 (11, 43)	.15	1.0
Age group distribution (%) among patients with non-IDU-SA (years)								
Mean	46 (44, 48)	45 (43, 47)	45 (43, 47)	45 (43, 47)	46 (45, 48)	47 (46, 49)	**.015**	0.16
15-34	15 (11, 21)	24 (18, 31)	21 (15, 27)	27 (21, 34)	21 (16, 27)	17 (13, 23)	.29	–0.22
35-54	58 (51, 65)	44 (37, 51)	50 (43, 58)	42 (35, 50)	44 (38, 51)	48 (41, 55)	.06	–0.51
55-64	27 (21, 34)	32 (25, 39)	29 (23, 36)	31 (24, 39)	35 (29, 42)	34 (28, 41)	**.004**	0.73
Race distribution (%) among patients with IDU-SA								
White	56 (30, 80)	50 (26, 74)	63 (38, 82)	78 (57, 91)	57 (35, 76)	58 (39, 75)	.11	1.2
Black	37 (16, 65)	28 (11, 55)	26 (10, 50)	8 (2, 26)	22 (9, 43)	23 (11, 43)	.38	–0.5
Hispanic	–	14 (3, 41)	12 (3, 37)	10 (2, 31)	17 (6, 43)	12 (4, 30)	.21	0.6
Asian/Pacific Islander	–	–	–	4 (1, 26)	4 (1, 25)	–	.20	0.1
Native American	–	–	–	–	–	4 (1, 23)	.18	0.2
Other	7 (1, 35)	8 (1, 40)	–	–	–	4 (1, 23)	.50	-0.2

Abbreviations: IDU-SA, injection drug use-related SAA; SA, septic arthritis of the foot and ankle.
